# Improving the experience of older people with colorectal and breast cancer in patient‐centred cancer care pathways using experience‐based co‐design

**DOI:** 10.1111/hex.13189

**Published:** 2021-01-13

**Authors:** Albine Moser, Inge Melchior, Marja Veenstra, Esther Stoffers, Elvira Derks, Kon‐Siong Jie

**Affiliations:** ^1^ Research Centre for Autonomy and Participation of Chronically Ill People Zuyd University of Applied Sciences Heerlen The Netherlands; ^2^ Department of Family Medicine CAPHRI Maastricht University Maastricht The Netherlands; ^3^ Department of Internal Medicine Zuyderland Medical Centre Sittard The Netherlands; ^4^ Research Centre for Integrative Patient Centred Health Care Zuyd University of Applied Sciences Heerlen The Netherlands; ^5^ Burgerkracht Limburg (Citizin Power Limburg) Sittard The Netherlands; ^6^ Department of Quality Improvement Zuyderland Medical Centre Sittard The Netherlands

**Keywords:** breast cancer, cancer care pathways, caregivers, colorectal cancer, experience‐based co‐design, older cancer patients, patient and public involvement

## Abstract

**Background:**

Patient and public involvement (PPI) in quality improvement of oncological care pathways for older patients are rare.

**Objectives:**

Improve the care pathway experience of older cancer patients and explore lessons learned regarding how to engage this vulnerable group.

**Design:**

**Experience‐Based Co‐Design**. Setting and participants: Older cancer patients, their caregivers and healthcare professionals within colorectal and breast cancer care pathways.

Interventions: Co‐design quality improvement teams.

Main outcome measures: Colorectal cancer care pathway touchpoints were (a) availability of a contact person during diagnostic, treatment and aftercare phases; (b) collaboration between physicians and different hospital departments; (c) continuous relationship with same physician; (d) respectful treatment; (e) and information transfer with primary care. Breast cancer care pathway touchpoints were (a) comprehensive information package and information provision, (b) care planning based on patient preferences, (c) continuity of patient–professional relationship and (d) specialized care in case of vulnerability. Challenges related to PPI included (a) ability of older cancer patients to be reflective, critical and think at a collective level; (b) gaining support and commitment of professionals; (d) overcoming cultural differences and power inequalities; and (e) involving researchers and facilitators with appropriate expertise and position.

**Conclusion:**

This multidisciplinary quality improvement project revealed several challenges of PPI with older cancer patients and their caregivers. Research teams themselves need to assume the role of facilitator to enable meaningful PPI of older cancer patients.

**Patient or Public Contribution:**

Patient and caregiver representatives and advocates were involved in the design, conduct, analysis, interpretation of the data and preparation of this manuscript.

## 1 INTRODUCTION

The total number of patients with cancer is increasing, in part due to earlier diagnosis and improved cancer treatment.^1^ In the Netherlands, for example, the 5‐year cancer survival rate has increased from 45% in 2000 to 65% in 2018.^2^ Cancer patients are an ageing population: in 2019, half of all new cancer patients in the Netherlands was 70 years or older.^2^ Most cancer patients in the Netherlands receive their care through multidisciplinary oncological clinical care pathways. In these pathways, patients are treated by a multidisciplinary oncological team according to national guidelines, to improve coordination and continuity of patient‐centred care.^3^ Care pathways have traditionally been developed by healthcare specialists, from a disease‐based perspective.^4^

Patient involvement in the development of care pathways is increasing.^3^, ^5^ Patients have been acknowledged for their unique experiences that can contribute to research‐based quality improvement.^6^, ^7^ Funding bodies require patient and public involvement (PPI) such as in the UK National Institute for Health Research body (INVOLVE), the Patient‐Centered Outcomes Research Institute (PCORI) in the US or the National Framework of Consumer Involvement in Cancer Control in Australia.^8^ In this study, we define PPI as the contribution of patients and their family caregivers in improving healthcare services by active involvement in a range of activities that combine experiential and professional knowledge.^9^ In the Netherlands, approximately 200 disease‐specific patient organizations are united within the Dutch Patient Federation.^10^ Patients, then, assume the role of patient representatives, telling a collective story to bring forth their concerns. A large number of patients, however, do not organize themselves, such as older cancer patients.

To date, several reviews have been published on PPI of older people.^11^, ^12^, ^13^, ^14^, ^15^ Baldwin et al^15^ recommended matching older persons skills and motivations to the project and level of involvement. Schilling et al^14^ found that effective involvement could be supported by critical success factors such as building good (equal) relationships, facilitating communication and breaking down barriers to participation such as providing a thoughtful choice of location. There are reviews on PPI in cancer in general^16^, ^17^, ^18^, ^19^; however, older cancer patients have primarily been involved in oncological end‐of‐life research.^20^ Bombard et al^6^ conducted a systematic review with the objective to identify the strategies and contextual factors that enable optimal engagement of patients in the design, delivery and evaluation of health services. Only two out of the 48 studies investigated cancer services. Overall findings show that low‐level engagement, mainly consultative unidirectional feedback, had an impact on discrete products (eg policy and planning documents and governance). High‐level engagement, mainly partnership approaches or co‐design, had an impact on care process or structural outcomes (eg collaboration and mutual learning and negotiating for service change). One such partnership approach is experience‐based co‐design (EBCD): patients, family carers and professionals share their experiences, identify and agree on improvement priorities and work together to achieve them.^7^ EBCD has been applied successfully to improve several cancer services in acute care, including breast,^21^, ^22^ lung,^21^, ^22^, ^23^, ^24^ head and neck,^25^ and gynaecological cancer^22^; in acute and community care for adolescent and young adults with cancer^26^; and in community care in an outpatient oncology care centre.^27^ Two studies described palliative care of older patients with cancer in emergency care.^28^, ^29^ All these studies show that there remain substantial areas for improvement on areas such as communication and interpersonal skills, patient and caregiver information, intra‐ and interorganizational continuity of care, collaboration among (medical) specialists, age‐specific subjects including fertility, cancer‐specific subjects such as cosmetic concerns and service‐specific subjects including day care. Despite the increasing number of published studies on PPI using partnership approaches, currently, studies involving older people with cancer are limited,^30^, ^31^ despite older patients constituting a majority in current clinical practice.^14^, ^15^, ^32^ The aim of this study was twofold: first, to improve the cancer care pathway experience of older cancer patients and, second, to explore lessons learned regarding how to involve this vulnerable group.

## 2 METHODS

### 2.1 Design

We used a participatory approach, where PPI is applied as partnership approach using the experience‐based co‐design (EBCD) methodology.^7^, ^33^ In this qualitative study, the subjective experiences of older cancer patients, caregivers and healthcare professionals are the starting point for quality improvement.

### 2.2 Setting

The study was conducted at Zuyderland Medical Centre (MC) in Heerlen, one of the largest urban teaching hospitals in the Netherlands. This hospital is located in one of the most ageing regions in the Netherlands, where 21% of the population consists of people older than 65 years (17.4% is the Dutch average). In 2004, Zuyderland MC signed a manifest called ‘contract with society’ as a promise to invest more in patient‐centred health care.^34^ We chose the colorectal and breast cancer patient pathways because these are the most common cancers in the Netherlands and, accordingly, are major pathways at Zuyderland MC.^35^

### 2.3 Participants

In this study, there were three target groups: older people with colorectal or breast cancer (n = 24), their caregivers (n = 24) and healthcare professionals (n = 32) (Table 1). Inclusion criteria for older people with cancer were age 65 years or older, receiving treatment in the hospital's colorectal or breast cancer pathway (in the diagnostic, treatment or aftercare phase), a life expectancy of more than 1 year and somehow vulnerable, such as having a small social network. We did not apply specific exclusion criteria because of the participatory nature of this study. Older people with cancer were purposively sampled and approached by the nurse (case manager) or an oncologist. All older people with cancer were asked to identify a caregiver—their spouse, relative or a friend—most involved in their care. The mean age and age range of the older people with breast cancer and colorectal cancer was 71.9 [65‐80] and 73.9 [65‐88] yrs., respectively. In the colorectal group, eight partners, one son and three daughters participated, and in the breast cancer group, there were six partners, three daughters, one son, one brother and one friend.

**TABLE 1:** Patient, carers and healthcare professionals' characteristics

	Patients (total n = 24)		Carers (total n = 24)		Healthcare professionals (total n = 32)	
Oncological cancer pathway	Colorectal (n = 12)	Breast (n = 12)	Colorectal (n = 12)	Breast (n = 12)	Colorectal (n = 20)	Breast (n = 12)
Sex male/female	7/5	0/12	5/7	8/4	12/8	2/10
Age mean and [range]	71.9 [65‐80]	73.9 [65‐88]	64.3 [48‐70]	65.8 [40‐84]	38.9 [26‐58]	42.3 [28‐55]
Relationship/Professional background			8P, 1S, 3D	6P, 3D,1S, 1B, 1F	7Ph, 1 GP, 4N, 6 AMP, 2HC	3Ph, 1 GP, 2N, 4 AMP, 2 HC
Disease phase	10C, 2M	12C				
Treatment phase	3D, 4T, 5A	2D, 4T, 6A				
Treatment^a^	10 R, 2 PCT, 5 CT, 3 RT	6 BA, 5 BCT, 5 CT, 5 RT, 7 HT				

Abbreviations: A, aftercare; AMP, allied medical professional; B, Brother; BA, Breast amputation; BCT, Breast conserving therapy; C, curative; CT, chemotherapy; D, Daughter; D, diagnostic; F, Friend; GP, general practitioner; HC, home care nurse; HT, hormonal therapy; M, metastasized; N, nurse; P, partner; PCT, palliative chemotherapy; Ph, physicians; R, resection; RT, radiation therapy; S, Son; T, treatment.

^a^ More than 1 treatment is possible: for instance, BCT, RT, HT, CT.

Healthcare professionals included 12 physicians (oncologists, surgeons, radiotherapists and general practitioners), 10 nurses (clinical nurse specialists, nurse practitioners, nurses from various departments and home care nurses) and 10 allied medical professionals (physiotherapists, dieticians, pharmacists and psychologists). The single inclusion criterion was involvement in the colorectal or breast cancer care pathways. Excluded were those who were not mentioned by the patients in the interviews, as they were likely less significant in terms of the experience of older people with cancer.

### 2.4 The research team and PPI

**FIGURE 1:**
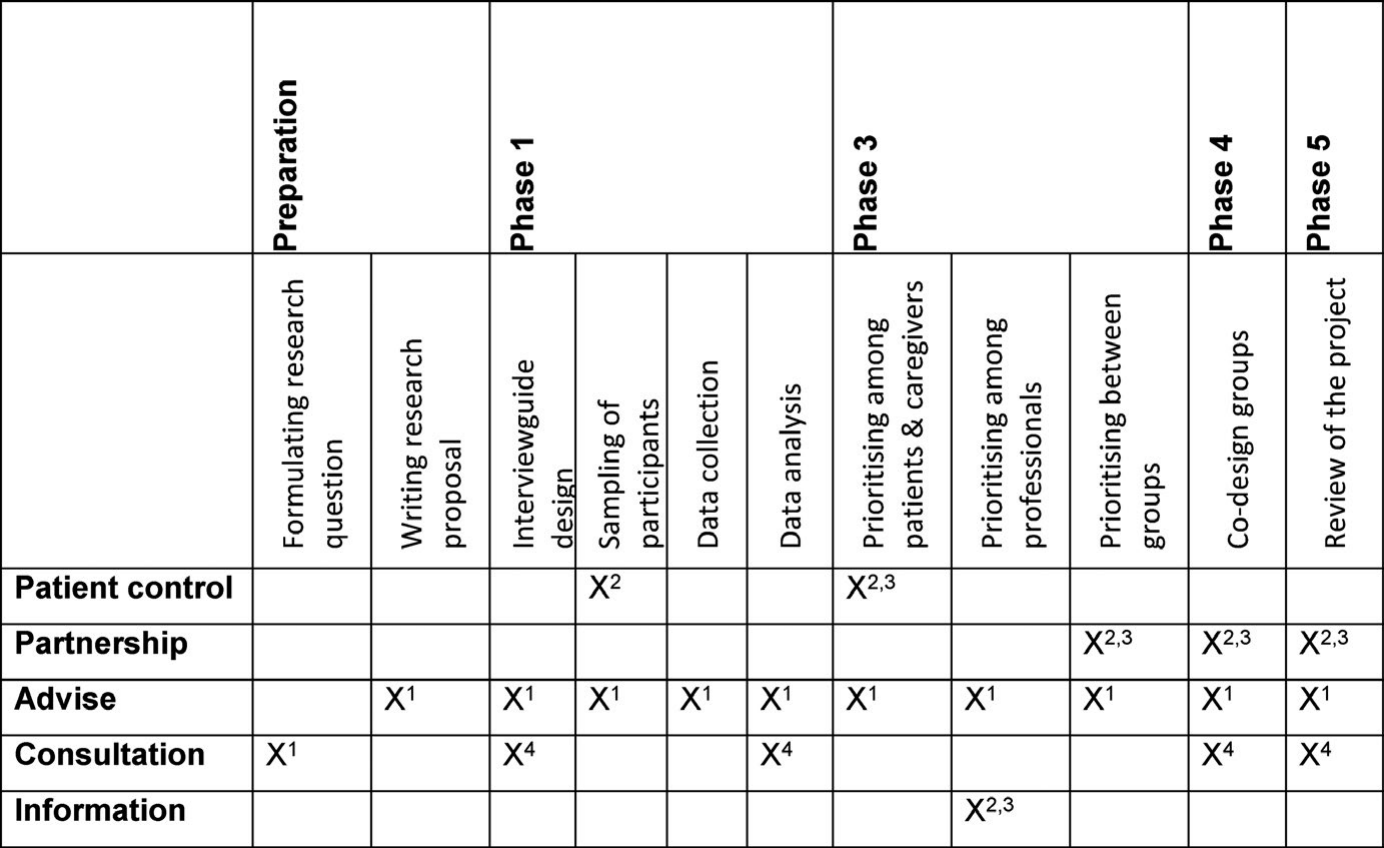
Patient and public involvement (as co‐designers), from Melchior et al ^33^ with permission from Dove Medical Press. Notes: 1 = staff members of the patient umbrella organization [Name organization], participating as patient advocates; 2 = cancer patients; 3 = caregivers of cancer patients; 4 = buddy of cancer patients; 5 = (former) patients with cancer, participating as patient representatives in the research team. Figure based on the participation ladder and theoretical thinking of Arnstein^36^

Our research team consisted of two project leaders: a nurse researcher (AM) and a haemato‐oncologist (KSJ), one patient and caregiver advocate (ED), two patient and caregiver representatives (MYV, ES) who were staff members of Zuyderland MC, a patient umbrella organization covering the southern region of the Netherlands, and two junior researchers (IM, AH). The patient and caregivers’ advocates and representatives acted as patient facilitators to support older cancer patients in their involvement as needed. In addition, also involved was an advisory board consisting of a representative of a National Comprehensive Cancer Organisation IKNL, a researcher with expertise in comorbidity and polypharmacy of older patients and primary care at Maastricht University, the chairwoman of the local Hospital Patient Organisation and a representative of the National Federation of Cancer Patient Organisations (NFK). We involved patient advocates in designing and managing the project, such as writing the research proposal, and designing and analysing the topic guides for the interviews. The ‘matrix of participation’ (Figure 1) was based on the ladder of Arnstein,^36^ which helped us to decide when to involve patients/caregivers or patient representatives.

### 2.5 Data collection and procedures

#### 2.5.1 Phase 1

**FIGURE 2:**
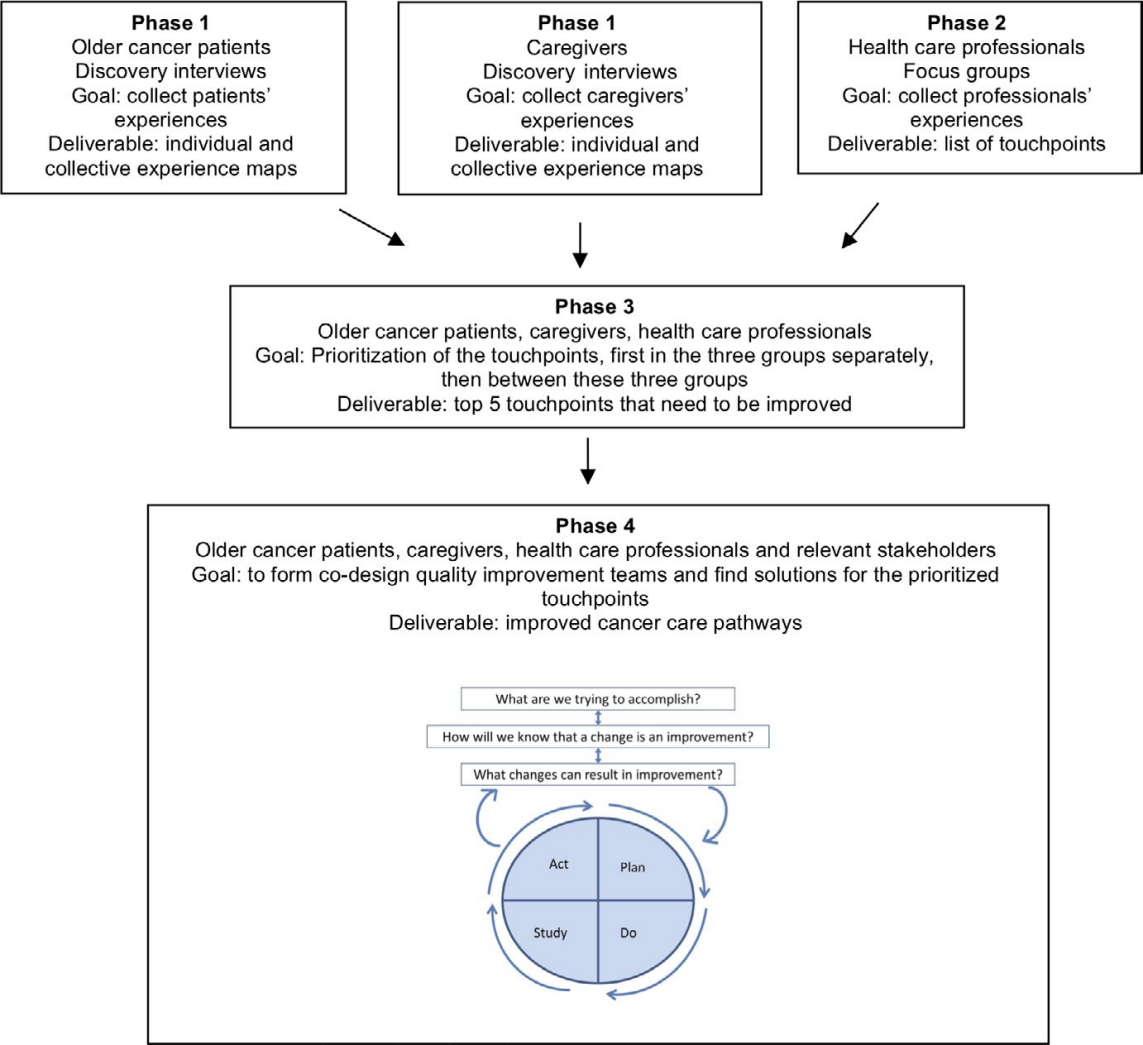
Experience‐based co‐design research process, from Melchior et al ^33^ with permission from Dove Medical Press

In Phase 1, we gathered the stories of the patients and their caregivers at the participants' homes, using in‐depth discovery interviews, each lasting between 45 and 120 minutes^7^ (Figure 2). The older people with cancer and their caregivers were interviewed separately if possible, to make sure that both voices were heard. However, in a few instances, the patient and their caregivers were interviewed simultaneously at the explicit wishes of the interviewees, to help the participants feel at ease. The interview started with a very open question: ‘Could you please tell me how you have experienced your cancer journey through the hospital?’ Touchpoints were subsequently further explored by more probing questions: ’What did you experience at that moment, what made that experience good/bad, and how did that make you feel?’ We used an interview guide (see File S1) to explore the following topics: comorbidity, polypharmacy, treatment, communication, provision of planning and information, and relationship with the general practitioner/home care. These topics were based on the requirements of the funding body but were also very often mentioned by patients and caregivers themselves. We paid particular attention to the provision of information in the diagnostic, treatment and aftercare phases to obtain the entire perspective. Reflective observational notes were made on how older people with cancer and caregivers related to their emotions, experiences and stories when they described their patient journey. The interviews were recorded on video or voice recorder, transcribed verbatim and subsequently interpreted into ‘experience maps’.^7^ An experience map is a visualization of the experiences. It captures the experiences, thoughts and actions of the interviewees. It allows us to understand their perspective. Experience maps help identify touchpoints and where and when there are opportunities for improvement.^7^ To make experience maps, we watched the videos and read the interviews to identify relevant experiences. Subsequently, we used these experiences to detect the touchpoints in the care pathways, for example relevant experience about side‐effects of chemotherapy we identified as touchpoint chemotherapy. Next, we ordered the experiences and resulting touchpoints in three phases: diagnosis, treatment and aftercare. These video and voice recordings were used in phase 3.

#### 2.5.2 Phase 2

In Phase 2, we gathered the positive and negative experiences of the healthcare professionals involved in the two care pathways by focus group discussions (Table 2). The central question posed was: ‘What, in your perception, can be improved for these older cancer patients and their caregivers?’ Questions were directed at the same domains as in the interviews with the patients and caregivers: starting with an open question and then focusing on the six aforementioned topics. Four focus group meetings were organized per clinical care pathway: one for the directly involved (eg case manager, haematologists), one for the indirectly involved professionals mostly by consultation or referral (eg, cardiologists, pulmonologists), one for professionals involved from outside the hospital (eg, home care, general practitioner) and one mixed. We presented the professionals' stories as a list of professional ‘touchpoints’, accompanied by quotations. We made field notes about the interactions between professionals from different disciplines to understand their differing and shared values and interactions.

**TABLE 2:** Participants per study phase^a^

	Colorectal cancer pathway			Breast cancer pathway		
	Patients (total n = 12)	Carers (total n = 12)	Healthcare professionals (total n = 20)	Patient (total n = 12)	Carers (total n = 12)	Healthcare professionals (total n = 12)
Phase 1	12	12		12	12	
Phase 2			20 (6Ph, 1GP, 3N, 5AMP, 3HC, 2QI)			12 (2Ph, 1GP, 4N, 3AMP, 2HC)
Phase 3 Prioritization separate groups	6	8	16 (4Ph, 1GP, 5N, 3 AMP, 3HC)	6	6	9 (2Ph, 1GP, 2N, 2AMP, 2HC)
Phase 3 Prioritization with all groups	9 9	9	12 (1Ph, 1GP, 3N, 2 AMP, 2HC, 3QI)	6	6	12 (2Ph, 1GP, 3N, 3AMP, 2HC, 1QI)
Phase 4	8	8	20 (4Ph, 1GP, 5N, 3HC, 5QI, 2PE)	4	4	9 (1Ph, 1GP, 2N, 2AMP, 2HC, 1Q)

Abbreviations: AMP, allied medical professional; GP, general practitioner; HC, home care nurse; N, nurse; PE, patient expert; Ph, physicians; QI, quality improvement fellows.

^a^ The total numbers of participants per phase could vary because the number of patients, carers and healthcare professionals in each phase was different.

#### 2.5.3 Phase 3

In Phase 3, older people with cancer, caregivers, and professionals prioritized the various touchpoints: first within each of the three groups separately (older people with cancer, caregivers, and professionals), and then with all groups together. Each prioritising meeting started with a presentation of the various touchpoints, illustrated by written or audio‐video taped quotations from the previous phases. Subsequently, one collective experience map was generated. After this, the participants prioritized the touchpoints which needed to be improved, individually with adhesive notes on the wall so that they became ‘collective’ touchpoints within the group. Each participant could assign three, two or one adhesive notes, assigning three to what they considered the most important touchpoint. The items with the most adhesive notes were prioritized. The last meeting ended with a consensus shortlist of touchpoints which needed to be improved. We also applied the three‐two‐one adhesive note system in the mixed groups, and the shortlist was composed of the items that received the most adhesive notes. The facilitators and barriers to patient engagement were noted in detail.

#### 2.5.4 Phase 4

In Phase 4, we formed five co‐design quality improvement teams involving older people with cancer and caregivers together with healthcare professionals to design and implement quality improvement in both pathways. Under the supervision of a facilitator and a patient advocate, each team explored the various layers of one of the touchpoints and its root cause. Subsequently, collectively, suitable solutions were considered, implemented or delegated and monitored using the Plan‐Do‐Study‐Act cycle.^37^ Quality improvement fellows from the hospital and managers with relevant positions facilitated the teams. In each quality improvement team, at least two older people with cancer and two caregivers participated. We made extensive notes on the challenges and facilitating factors in the co‐design quality improvement teams.

### 2.6 Data analysis

All interviews and focus group discussions were video‐ or audiotaped and transcribed verbatim. Field notes were written down during the observations of all meetings. We analysed all data using open and axial coding strategies and comparative analyses.^38^ The data were read to identify text passages relevant to the research question. Each relevant line of the interview data was coded using open codes that were often descriptions used by the respondents. Next, codes were grouped into subcategories and categories that best characterized the data collected. Throughout the analysis process, the codes, subcategories and categories were constantly compared and contrasted within and among the data. Memos were written about these codes and (sub) categories and of the analysis process itself. These were examined for links and connections to further the analysis. The analysis resulted in touchpoints, improvement priorities and outcomes and the four lessons learned. We derived categories grounded in the data, and also based on the six topics required by the funding body. Three members were involved in the analysis. In the event of different interpretations of text fragments and different codes assigned, the original quote was reviewed to capture the intended meaning. After discussing the meaning, an agreed‐on code was assigned. Qualitative data analysis software Nvivo (version 10 for Windows) was used to process the data. We stopped the analysis (and data collection) after we reached data saturation. We determined data saturation by the degree to which new data repeat what was expressed in previous data meaning that data were replicated in the interviews.^39^

### 2.7 Trustworthiness

We sought to safeguard credibility and transferability.^40^ Credibility was ensured using various kinds of data (cancer patients, caregivers and healthcare professionals), methods (discovery interviews, focus group discussions and field notes) and investigator triangulation (see research team section above). In addition, we member‐checked the transcript. Furthermore, ‘thick descriptions’ of the context of the particular patients, and the social settings in which the data were gathered, were used to inform other researchers about the extent to which the findings are transferable to other contexts.

### 2.8 Ethics approval and informed consent

The project was approved by the Ethics Commission of Zuyderland MC. Participants received information before the start of the study were allowed to ask questions and signed an informed consent form.

## 3 FINDINGS

We gained rich data and reached data saturation, with a sample size that is consistent with several other studies using EBCD.^26^, ^27^ The number and characteristics of participants and the background of the healthcare professionals involved (per study phase) are shown in Table 1 and Table 2. In the following subsection, we present the key touchpoints, improvement priorities and outcomes for each cancer care pathway and our reflection on four challenges of involving older people with cancer as equal partners.

### 3.1 Touchpoints, improvement priorities and outcomes: colorectal cancer care pathway

Older people with colorectal cancer, caregivers and healthcare professionals identified and prioritized several touchpoints. Table 3 illustrates the perceived key touchpoints per group.

**TABLE 3:** Overview of key touchpoints per cancer care pathway and group

	Caregivers	Professionals
Colorectal cancer patients		
Follow‐up support/contact person	Contact/information transfer with family physician	Contact person in treatment phase
Collaboration between physicians	Collaboration between physicians and different hospital departments	Visibility of contact person to other healthcare professionals
Continuous relationship with the same physician	Skills professionals	Respectful treatment
Contact with family physician	Follow‐up in hospital	Information transfer with home care
Respectful treatment	Information transfer with home care	Responsible physician
Breast cancer patients		
Guidance for informal caregivers	Emergency services and contact person in case of emergency	Information provision about treatment, just‐in‐time across the pathway, and information material
Information provision about the treatment and practical information	Information provision during breaking bad news and tissue expander	Psychosocial aftercare
Continuity of patient–professional relationship	Waiting time during the day of surgery	Continuity of patient–professional relationship
Manner of approaching	Skills healthcare professionals	Care planning based on preferences of patients
Information about life expectancy	Guidance informal caregivers	Specialized care in case of vulnerability

The prioritized collective touchpoints were (a) availability of a contact person during diagnostic, treatment and aftercare phases; (b) collaboration between physicians and different hospital departments; (c) continuous relationship with the same physician; (d) respectful treatment; and (e) information transfer with primary care.

#### 3.1.1 Availability of a contact person during diagnostic, treatment and aftercare

This touchpoint received the highest prioritization, especially from older people with cancer and healthcare professionals. Older people with cancer reported a lack of availability for out‐of‐hours consultations. Older people with cancer and caregivers felt that they did not receive necessary information in a timely fashion throughout their treatment trajectory, including what to expect during their patient journey. Tailored and person‐centred provision of information was important to all older people with colorectal cancer.

Older people with cancer and healthcare professionals, especially nurses, prioritized the role of nurses as case managers throughout the entire cancer pathway, from the moment of diagnosis to aftercare. They felt that continuity of care was lacking, and often, older people with colorectal cancer felt unsupported and needed to find solutions without assistance.

Every time there was another professional on the phone. Or in some instances, they [nurse or doctor] called back. They did it professionally, nothing to say about this. However, there was never a professional that I could contact directly. At the beginning, you have to deal with a lot of new things […] I felt left alone. (Patient 12)

#### 3.1.2 Collaboration between physicians from different hospital departments

This touchpoint was especially relevant to older people with colorectal cancer and their caregivers. They experienced that physicians from different departments insufficiently informed each other about relevant health issues. They desired a physician who had an overview of all treatment modalities. They expressed the need to be treated as a whole as they felt physicians were uninformed about them, nor did they consult other physicians about the comorbid health problems.

It seems that there is nowhere a connecting path. There is a street for cancer, a street for cardiology, a street for urology but no connecting paths, no doors that can be crossed. The doctors in the cancer street do not speak to the doctors in the cardiology street. There are no connecting paths. And you have the feeling that every department and physician is superior to the other and the others to be subordinate. (Caregiver 3)

#### 3.1.3 Continuous relationship with the same physician

Many older people with colorectal cancer felt a continuous relationship with their physician was lacking. Although they were aware that certain medical specialists have a particular role at certain times in the illness trajectory, establishing a relationship and trust was important to them in moments they felt vulnerable.

After surgery, we had a meeting with the surgeon. He was not there but a colleague. We do not know why. The surgeon who did the operation never visited me at the ward. The colleague who substituted the surgeon was quite focused. He asked if I had problems. He did not even inspect my abdomen at all, nor my colostomy. How can I trust him? (Patient 10)

#### 3.1.4 Respectful treatment

Older people with colorectal cancer and healthcare professionals experienced a lack of ‘soft people skills’. They perceived that healthcare professionals sometimes lacked interpersonal skills such as an empathic attitude, time to listen or utilising the opportunity to build a good relationship. They mentioned unfriendly communication styles because of work pressure and time constraints.

He was in a hurry. But then I think why don't you prepare yourself by looking in my patient file before you start a conversation with me (Patient 3)

He is in front of the computer, in which all my files can be found. But the human being who was in front of him, I don't think he can recall that (Patient 12)

#### 3.1.5 Information transfer with primary care

Older people with colorectal cancer, caregivers and healthcare professionals experienced a lack of needed information during the transfer from and to primary care, especially to family physicians and home care nurses. Older people with colorectal cancer and caregivers missed the transfer of information at the time of discharge. In certain cases, the care capacities in the home environment were insufficiently taken into account.

What we do is that we call the family practitioner: “Your patient has had surgery and will be discharged in a few days.” But we do not have a well‐designed procedure or discharge‐service. Because, imagine an older patient going home with a wound, a colostomy, weakened and what now? What kind of care? Somehow, we here in the hospital, let them down. (Nurse 1)

Healthcare professionals expressed the desire to obtain patient‐specific context information before making medical decisions, such as the availability of the social network or do‐not‐resuscitate orders.

Based on the prioritization, four co‐design quality improvement teams were formed. The prioritized touchpoints ‘collaboration between physicians and different hospital departments’ and ‘continuous relationship with the same physician’ were combined in one co‐design quality improvement team because the focus was similar. In total, 14 co‐design quality improvement team meetings were held, and together they worked on 16 outcomes, which were incorporated into care processes (Table 4).

**TABLE 4:** Prioritization, co‐design quality improvement teams and outcomes per cancer care pathway

Prioritization of collective touchpoints	Focus on quality improvement	Outcomes
Colorectal cancer patients		
Availability of contact person during diagnostic, treatment and aftercare phases	Continuity of care and primary contact person	1. Information in the patient‐folder ‘24 h accessibility’ was added about whom (case manager, unit, emergency service etc) to call during the day, evenings and weekends. 2. Provision of tailored information based on the needs and wishes of elder people and caregivers. The development of the website is underway. 3. The role of nurses (case managers) as primary contact point was advanced from the diagnoses phase throughout the cancer care pathway encompassing diagnosis, treatment and aftercare. If needed, medical specialists will be directly consulted.
Collaboration between physicians and different hospital departments and continuous relationship with the same physician	Primary responsible physician	1. Conscious and visible registration in the electronic patient record of the identity of the primary treating physician in a specific time. 2. Continuous documentation of interprofessional and multidisciplinary consultation in the electronic patient record. 3. Continuous evaluation times between older people with cancer and nurse (case manager) to review the above‐mentioned agreement.
Respectful treatment	Interpersonal skills and service development	1. Establishment of shared outpatient clinics between surgeon and oncologist to support access to the same doctor. 2. Current information for older people with cancer and caregiver about the waiting time in the outpatient clinic. 3. Implementation of a working process that allows the doctor to prepare the patient case beforehand and not during the outpatient contact. 4. Establishment of a rapid‐response work procedure for nurses at the oncology unit to respond timely to emergencies 5. Weekly lunch meeting at the oncology unit to reflect on current bottlenecks and facilitate instant quality improvements. 6. Implementation of two fixed time slots for discharge per day to appoint precise pick‐up times for caregivers. 7. Involvement of caregivers in the intake procedure at the oncology unit to provide relevant background information.
Information transfer with primary care	Information transfer from and to primary care	1. The discharge information of the cancer unit is also forwarded to the home care nurse (next to family physician). 2. Introduction of a checklist for nurses in addition to discharge information: older people with cancer and caregivers will receive a copy of the checklist 3. Introduction of an information sheet where family physicians provide patient‐relevant information (eg Do not resuscitate wish) at the point of admission. This information is used in the multidisciplinary team meetings on individualized treatment decisions.
Breast cancer patients		
Comprehensive information package and information provision Care planning based on preferences of patients	Breaking bad news conversation and tailored information provision within shared decision making throughout the pathway	1. Definition of personalized care and treatment goals during the breaking bad news conversation. 2. Implementation and roll‐out of shared decision making using the Ask Share Know approach, in all decisions in breast cancer care next to treatment decisions.

### 3.2 Touchpoints, improvement priorities and outcomes: breast cancer pathway

Older people with breast cancer, caregivers and healthcare professionals identified and prioritized several touchpoints (Table 3).

The collective touchpoints were (a) comprehensive information package and information provision, (b) care planning based on patient preferences, (c) continuity of the patient–professional relationship and (d) specialized care in case of vulnerability.

#### 3.2.1 Comprehensive information package and information provision

Older people with breast cancer, caregivers and professionals experienced that information material was not always up‐to‐date, and some parts were missing, such as information regarding aftercare. Family members experienced that they sometimes received information twice or some necessary information, not at all. In addition, professionals in the care pathway provided individual information packages as stand‐alone packages. Information was provided in multiple leaflets. There was a need to streamline the information with respect to content and timing, for themselves and the caregivers.

Every subsection in the care pathway writes his own information flyer. They are not integrating the content, format and style. We lack a folder where information flyers can be added for individualized pathways. (Nurse practitioner 2)

#### 3.2.2 Care planning based on patient preferences

All three groups experienced problems related to patient preference. Especially, women with breast cancer missed practical information such as where they can buy bras where the shop employee is respectful and trained to serve women like them. Older breast cancer patients missed a tailor‐made, patient‐centred approach to participate in decision making fully; they especially missed treatment information about different possible treatments.

They [professionals] do not see me as a person. Patients are not always involved in decisions about the treatment. I was not informed about the pros and cons of the treatments. If I know in advance what the pros and cons are of available treatments, I can make better decisions and whether the treatment makes sense in my personal situation. (Patient 16)

Caregivers missed information about specific treatments, such as tissue expansion, and side‐effects. They were unsatisfied with the breaking bad news conversation and how little the living conditions of their loved ones were part of this conversation.

They told us about treatment with medication or surgery – very fast. Did I understand that there were only two options – turning left or turning right. We did not get any information about survival. It was ‘this is the best option, go home, think about it, and in 2 weeks you come back with a decision’. I had expected a kind of protocol or algorithm. The context of my mother, who turns 85 next month, is different, the decision is different. (Caregiver 23)

Professionals were mostly concerned about providing the necessary information about the treatment they provided, and they perceived as relevant. Professionals said that it was hard to provide patient‐centred information just‐in‐time across the pathway, especially for older people who suffered from multi‐morbidity. They perceived that older people with breast cancer did not get a comprehensive care plan which took into account their personal preferences and needs.

#### 3.2.3 Continuity of patient–professional relationship

The patients and professionals stressed the importance of having the same healthcare professional during their entire treatment or informing the patient beforehand when they would see another professional during their patient journey.

A personal relationship, because the doctor knows more about you as a person, than what can be found on the electronic health record. That gives you more confidence, because you are sure that you are being treated in a way that suits your personal situation. (Patient 15)

In a multidisciplinary setting, the professionals stressed that it was inevitable that the patient would see different physicians during their patient journey (ie a surgeon, a radiotherapist or an oncologist).

#### 3.2.4 Specialized care in case of vulnerability

Professionals especially experienced a lack of care for the vulnerable older patient with cancer. They acknowledged that they increasingly were treating a new patient group aged over 80, and missed dedicated geriatric services.

Professional 1: ‘Now we see a group of women who are 80 ‐ 85 years old. We see them much more. This has changed’. (Surgeon 3)

Professional 2: ‘The geriatrician is automatically consulted for people with lung cancer, not for breast cancer. The geriatrician has also an important role, but people with breast cancer stay very short at the hospital. The transfer to the home setting should be well organized. The geriatrician can identify risk factors and prevent typical geriatric problems’. (Nurse practitioner 3)

Professional 1: ‘Yes, when those women go home, many problems occur. Yes, it goes wrong. It often goes wrong. A screening is missing’. (Surgeon 3)

In the breast cancer pathway, the prioritized ‘collective touchpoints comprehensive information package and information provision’ and ‘care planning based on preferences of patients’ were combined in one co‐design quality improvement team. Older people with cancer, caregivers and professionals perceived that these two areas for improvement were interrelated. For the touchpoints ‘continuity of patient–professional relationship’ and ‘specialized care in case of vulnerability’, no co‐design quality improvement teams were organized because at that time the breast cancer pathway‐team lacked sufficient manpower, time and suffered from heavy workload to establish another quality improvement team. In total, three co‐design quality improvement team meetings were held, and together they worked on two outcomes, which were incorporated into care processes (Table 4).

### 3.3 Reflection on main challenges

#### 3.3.1 The ability of older people with cancer to be reflective, critical and think at a collective level

It was striking to notice how the older people with cancer struggled to think at a collective level, to adopt a helicopter view and to go beyond their personal experiences. Several older people continued to share their personal experiences, without being able to translate them into general arguments, which impeded the progress of the meeting. Although we thought that giving them preparatory homework in advance of the co‐design quality improvement teams might better prepare them, their unfamiliarity with reading documents made them lag even further behind the professionals. Eventually, the facilitator decided to prepare the patients face‐to‐face without written documents, half an hour before the start of the meeting with the professionals.

#### 3.3.2 Gaining support and commitment of the professionals

The approval and cooperation of the professionals were needed to make PPI a success. Although several nurses and the general practitioners were supportive from the beginning, the group of physicians were especially difficult to reach. They were quite sceptical about qualitative research in general (what valuable information can interviews and a few group talks really yield?), and they seemed quite doubtful about the idea of discussing the quality of care with non‐professionals, afraid that they would come up with unrealistic demands. Because of their limited time available, some physicians sometimes sent their assistants to represent them in the prioritization and implementation phases. The majority of other professionals, however, participated throughout the whole project. Finally, the physicians did not seem convinced that older people with cancer could design original solutions that the professionals had never considered.

#### 3.3.3 Overcoming cultural differences and power inequalities

The facilitators, the patient and caregivers advocate (ED) and representatives (MYH, ES), as members of our project team and trained in PPI, played an essential mediating role. They set the new rules for interaction and created space for older people with cancer and caregivers in the discussions and ensured the use of a common language. Maintaining physical closeness during the meetings, they could easily create this safe space for older people and their caregivers. The social hierarchies between older people with cancer, caregivers and professionals were sometimes difficult to circumvent. Scheduling of the meetings was centred around the professionals’ schedules, at the end of their working day. The older people with cancer were expected to adapt in terms of location and time. As a result, those who depended on their working relatives for transport were thus sometimes unable to participate. Furthermore, the meetings took place in the occupational setting of the professionals, their ‘natural habitat’. This inequality between participants was further reinforced when the professionals wore their white uniforms. In the co‐design quality improvement teams, professionals found it difficult to avoid predetermined solutions, rather than creating improved methods with the patients. When the team discussed the lack of a central contact point for the patients, the professionals initially saw no need to discuss this point anew with the patients, as they already had found a predetermined solution: assigning a dedicated team of nurses as a central contact point. The patients’ solution, however, was economically more advantageous, as they just wanted a business card with phone numbers for consultation. This was surprising for the professionals, as they had never understood the ‘real’ problem from the older people's perspective, although they had been convinced they did.

#### 3.3.4 Involving researchers and facilitators with the appropriate expertise and position

Another challenge was to position ourselves as project group members strategically. We acted as the advocates of older people with cancer and their caregivers, aimed at creating an environment that supported involvement. The anthropologist, being the first point of contact between the older people and caregivers and the project team, had the main advantage of being considered relatively independent of the hospital environment. In this way, she may have earned the confidence from the older people to share their stories honestly. Establishing a personal relationship during the interviews helped to create a commitment to involvement during the rest of the project, as they felt they had become part of a community. Our haemato‐oncologist, being both an advocate of PPI and a physician and insider in the hospital, acted as a liaison to his fellow healthcare professionals. Another crucial role was reserved for the two patient advocates, who were seen as independent from the hospital: they could represent the patients and propose unorthodox ideas without being socially and hierarchically restricted. A final important facilitator in our project was our patient and caregivers advocate (ED), a former nurse who worked as an employee of the Patient Service desk. As an insider at the hospital, yet a critical one, and having been trained in PPI, she was very successful in binding both the healthcare professionals and ‘taking over’ the anthropologist's personal relationships with the patients and caregivers. The traditional hierarchies between professionals could hardly be circumvented, and to attain PPI of older cancer patients, we needed to use our backgrounds strategically and adapt ourselves to meet the norms of the traditional power holders.

## 4 DISCUSSION AND CONCLUSION

The aim of this study was twofold: to improve the cancer care pathway experience of older people with cancer and second, to explore lessons learned regarding how to involve this vulnerable group. Collective touchpoints in the colorectal cancer care pathway were availability of a contact person during diagnostic, treatment and aftercare phases, collaboration between physicians and different hospital departments, continuous relationship with the same physician, respectful treatment and information transfer with primary care. Critical touchpoints in the breast cancer care pathway were provision of comprehensive information packages and information, care planning based on patient preference, continuity of patient–professional relationship and specialized care in case of vulnerability. The challenges were the ability of older people with cancer to be reflective, critical and think at a collective level, gaining the support and commitment of the professionals, overcoming cultural differences and power inequalities, and involving the researchers and facilitators with the appropriate expertise and position.

Comparing the prioritization of collective touchpoints of older people with cancer and the topics requested by the funding body, it seems that they do not differ substantially from previous studies.^21^, ^24^, ^25^, ^26^, ^27^ Agreements concerned the areas of communication, decision making and interpersonal skills, patient and caregiver information, continuity of care and collaboration among (medical) specialists. Although our quality improvement suggestions were incorporated into the care processes of the cancer care pathways, further research is needed to evaluate the long‐term effects. It seems that older people with cancer and caregivers have generic concerns, which is surprising because we would have expected age‐specific and cancer type‐specific areas for quality improvement. What came to the fore was that information at the breaking news conversation should include age‐relevant facts such as survival rates. However, in the co‐design quality improvement teams, we ensured that setting specific quality improvement actions addressed these generic concerns.

Notably, the question of inequality in partnerships did not seem to be a problem for older people with cancer. They considered much more important than equality in the teams was the extent to which they were able to form a team and a community feeling based on mutual respect. Our participants deemed it essential that they identified each other, realized that they needed each other and that they had a common goal on which to focus. We should, therefore, focus more on discovering the individual personal and professional strengths of all participants to better ‘match older people's skills, expertise and motivations to appropriate roles’ and use those strengths at the right place and time in research projects.^15^ We asked ourselves in which stages would it be most meaningful to involve older people with cancer, what kinds of people in which process phase and how they could participate. While older people or cancer patients are often involved in one stage of research,^11^, ^15^, ^19^ we succeeded in maintaining continuity of involvement.

We realized that the older people with cancer struggled to think at a collective level, to adopt a helicopter view and to go beyond their personal experiences. This, however, is absolutely not meant in a blaming way. Rather, at the time of the study they were all cancer patients who were actually receiving cancer treatment. They were in a vulnerable position and occupied by their own personal experience and life situation. For phases 3 and 4, we therefore recommend researchers to engage a combination of older people with cancer, older people beyond the active treatment phase and/or representatives of patient organizations in advance to identify the skills, experience and personal attributes that they will need to have to fully make use of the potential of PPI. It also helps to build a relationship, reflect on roles, responsibilities and expectations and to have a dialogue on their participation preferences. Our findings show that getting a close match between the requirements of the task and the individual performing the task is really important.

Although we wanted to create a mindset of equal partnership, which is an enabler of PPI,^15^ we did not manage to escape the disciplinary power of the professionals. We realized along the way that we researchers had to adapt to the social norms of the healthcare professionals, as their involvement—being the traditional power holders—was essential to ensure PPI success. Although PPI has given more power to older people with cancer, its success depends entirely on the commitment of the professionals as the traditional power holders. The Dutch National Fund for Health Research, ZonMw, has recently made PPI a prerequisite for funding (ZonMw, 2019). The external motivation of PPI, however, might result in insincere assurances.^15^

What we did notice is that once professionals had become involved in the prioritization phase (and beyond), they were less likely to drop out along the way. Hearing and seeing the emotions of the older people with cancer and their caregivers on video or in voice recordings^7^ made them committed witnesses. Professionals, mostly nurses, who already wanted to initiate change among hospital management, now have the patients' and caregivers' stories as testimony. In this way, these stories enabled them to become change agents for better care.^41^

Another limitation is that in the relatively short timeframe of the project we were not able to set up all co‐design teams. In the breast cancer pathway for two touchpoints, no co‐design quality improvement teams were organized. This limitation has impeded observing the quality improvement cycles and studying facilitators and barriers in this specific moment in the implementation of quality improvement. We also did not further explore how to deal with staffing and high workload and its impact on the EBCD approach.

Although we wanted to involve a group that is vulnerable, hard to reach and underrepresented,^33^ older people with cancer such as those aged over 90 years, those with multiple disabilities or the very ill, did not want to participate or were simply unable to do so. Their voices remain unheard.

In conclusion, the project revealed several challenges of PPI for older people with cancer, caregivers and professionals in multidisciplinary quality improvement. Future initiators of participatory research projects should not let the inherent inequality among them detract from implementing PPI to improve the quality of cancer care pathways. Instead, they should strive for meaningful PPI, to better utilize the older people's personal skills, expertise and motivations for appropriate roles. Research teams (which should include patient and caregiver advocates and/or representatives) need to take the role of facilitator to enable meaningful PPI of older cancer patients and caregivers.

## CONFLICT OF INTEREST

The authors declare that there is no conflict of interest.

## AUTHORS' CONTRIBUTIONS

AM and KSJ had significant involvement in the design, analysis and interpretation of data and writing the manuscript, IM was involved in the acquisition, execution, analysis, interpretation of data and writing the manuscript; MV, ES and ED contributed as patient and caregivers representatives (MV,ES) and patient advocate (ED) and had a major role in the execution of the study, analysis and interpretation of data. All the named authors agree to take accountability for the integrity and accuracy of the work and have read and approved the final manuscript.

## Supporting information

File S1

## Data Availability

Data available on request due to privacy/ethical restrictions.
