# Supplementary material for: Improving the experience of older people with colorectal and breast cancer in patient‐centred cancer care pathways using experience‐based co‐design
Source: Health Expect. 2021 Jan 13;24(2):478–90. doi: 10.1111/hex.13189 (PMC8077111; doi:10.1111/hex.13189)
Supplement: Supplementary file 1 — File S1 [file HEX-24-478-s001.pdf]

Supplement Box 1 Interview guide

Journey through the cancer care pathway
Could you please tell me how you have experienced your cancer journey through the hospital?

Examples of probes
• The first complaints
• The general practitioner and referral
• Undergoing medical examinations
• Breaking bad news
• The treatment
• The operation
• Consultation with the medical specialist
• Aftercare
• Interaction with the general practitioner and home care

Questioning key moments
What were the important moments for you?

Probes for each key moment
• What have you experienced? [experience]
• What made the experience nice/unpleasant? And why? [evaluation/reflection]
• What did that do to you? How did it make you feel? [feeling]

Questions on the experiences and feelings specific to
1. Provision of information/education
2. Treatment and communication
3. Planning
4. Comorbidity
5. Polypharmacy
6. Relationship with the general practitioner/home care

Examples of probes
<u>Ad 1 – Provision of information/education</u>
For the cancer patient
• Was the information you received sufficient and clear?
• Every time you left the consultation room, did you feel that you knew what to expect next?
• Has the care provider explained well what your concerns/complaints could indicate?
• Did you know where to get help? Was it clear to whom you could turn with specific questions?
• Did the care provider guide you well to familiarize you with 'the world of the cancer patient'? For example, did he/ she refer you to a 'Hospitality House' for cancer patients?

For the informal caregiver
• Did you receive sufficient information/advice regarding your options for combining informal care with your work?
• Did you receive sufficient information/advice regarding your options for requesting additional services (resources)?

Ad 2 – Treatment and communication
For the cancer patient
• Did you have the feeling that the care providers empathized with you? What did that mean to you?
• How did you experience closeness, inequality, and mutual respect? Did you experience unpleasant moments?
• Do you feel that the care providers listened to you? Do you have an example?
• How did you experience discussing the treatment? Did the doctor/nurse give you multiple treatment options or tell you which treatment you would receive?
• Did you experience the language of the care providers as understandable? Did they use lay language?
• Did you feel that you could address your questions to the doctors or other staff? Did you ask for clarification?
• Did you experience the communication of the care providers as consistent over time? Did they repeatedly provide the same information, or did it change?

For the informal caregiver
•Did you receive information relevant to you? Were you informed about the treatment options? If no, why not? What was your role towards medical specialists, nurses or other hospital staff?
• Were sufficient provisions made for you as an informal caregiver? What amenities were important to you? Did you feel sufficiently informed?

Ad 3 – Planning
For the cancer patient
• How did you experience treatment planning? Too stressful? Too fast or too slow? Were you well informed or surprised?
• How did that feel for you?

For the informal caregiver
• What kind of planning issues arose for you, and how did you deal with them? Did you feel supported by hospital staff?

Ad 4 – Comorbidity
For the cancer patient
• Do (did) you have symptoms of other diseases that occurred simultaneously with cancer? So yes, which symptoms?
• Were you well informed in advance of the symptoms that you could expect?
• How did you experience the provision of information about these symptoms? Were you well prepared?
• How did you experience the collaboration among medical specialists?

For the informal caregiver
• How well were you prepared to deal with other diseases?
• What kind of support did you receive (or not) from the staff?

<u>Ad 5 – Polypharmacy</u>
For the cancer patient
• Did you receive new medication? How was that experience?
• Was it clear on how to use the new medication? Was it clear how to use the new medication in relation to the medication you were already taking?
• How did you experience the use of the different medication? Were there any uncertainties? Did you get sufficient information?
• Did you experience side-effects using the medication? Were you well supported through that? By whom?

For the informal caregiver
• How well were you prepared to provide medication support? What was your caregiving role?
• What kind of support (or not) did you receive from the staff or (community) pharmacy?

<u>Ad 6 – Relationship with the general practitioner/home care</u>
For the cancer patient
• How did you experience the collaboration between hospital care providers and the general practitioner/community nurse? What went well? What needs to be improved?
• How did you experience the collaboration between the aftercare specialists? Were they well aware of each other's statements and agreements? Did they inform your general practitioner?
• If they contradicted each other or did not work well together, how did that feel for you?
• How did you experience the transition from the general practitioner to the hospital, and discharge from hospital to home care?

For the informal caregiver
• How were you involved in the aftercare provided by the general practitioner and/or community nurse? Did you receive support from the discharge nurse?
• What kind of support did you receive from them? Were your needs met? If not, why not?

Ending of the interview
[Finish again with a very open question]
"What is the most positive and most negative memory for you since you arrived at the hospital? With what are you very happy and not happy? What should we definitely pay attention to if we want to improve the care for the older cancer patient?"

Are there persons or moments we have not talked about that are or have been important to you?
